# The Radiobiological Effects of Proton Beam Therapy: Impact on DNA Damage and Repair

**DOI:** 10.3390/cancers11070946

**Published:** 2019-07-05

**Authors:** Eirini Terpsi Vitti, Jason L Parsons

**Affiliations:** Cancer Research Centre, Department of Molecular and Clinical Cancer Medicine, University of Liverpool, Liverpool L3 9TA, UK

**Keywords:** DNA damage, DNA repair, proton beam therapy, radiobiology

## Abstract

Proton beam therapy (PBT) offers significant benefit over conventional (photon) radiotherapy for the treatment of a number of different human cancers, largely due to the physical characteristics. In particular, the low entrance dose and maximum energy deposition in depth at a well-defined region, the Bragg peak, can spare irradiation of proximal healthy tissues and organs at risk when compared to conventional radiotherapy using high-energy photons. However, there are still biological uncertainties reflected in the relative biological effectiveness that varies along the track of the proton beam as a consequence of the increases in linear energy transfer (LET). Furthermore, the spectrum of DNA damage induced by protons, particularly the generation of complex DNA damage (CDD) at high-LET regions of the distal edge of the Bragg peak, and the specific DNA repair pathways dependent on their repair are not entirely understood. This knowledge is essential in understanding the biological impact of protons on tumor cells, and ultimately in devising optimal therapeutic strategies employing PBT for greater clinical impact and patient benefit. Here, we provide an up-to-date review on the radiobiological effects of PBT versus photon radiotherapy in cells, particularly in the context of DNA damage. We also review the DNA repair pathways that are essential in the cellular response to PBT, with a specific focus on the signaling and processing of CDD induced by high-LET protons.

## 1. Introduction

**Figure 1:**
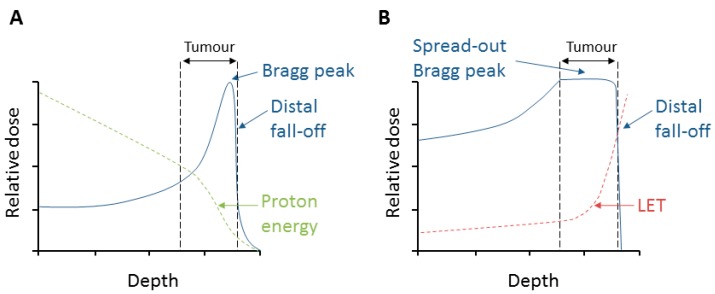
Depth–dose distribution of protons and relationship to energy and linear energy transfer (LET). (**A**) An unmodulated (pristine) Bragg peak produced by a proton beam. (**B**) Spread-out Bragg peak (SOBP) from several modulated proton beams.

Since its first application in the 1950s, proton beam therapy (PBT) is gaining ground in radiation oncology thanks to its radiobiological and physical advantages over photon radiotherapy [1]. Proton beams are characterized by a low entrance dose, whereby the protons lose energy along the track and just before they stop, the dose peaks in depth at a narrow and well-defined range called the Bragg peak (Figure 1A). The energy deposition drops rapidly shortly after the peak at the distal fall-off. This spares the surrounding tissue and organs at risk (OARs) in close proximity to the tumor being treated. A combination of beams with different initial energies can produce a wider peak, the so-called spread-out Bragg peak (SOBP), allowing the irradiation of larger target tumor volumes [2] (Figure 1B). However, as the protons slow down and lose energy further, their linear energy transfer (LET) increases and becomes maximal in the distal fall-off of the Bragg peak.

As of now, there are 70 operative facilities worldwide for PBT and 42 under construction according to the Particle Therapy Co-Operative Group (https://www.ptcog.ch), with 150,000 patients receiving PBT treatment. Despite over 60 years of therapeutic use of protons, there are several uncertainties regarding the relative biological effectiveness (RBE) of the proton beam along the track, particularly throughout the SOBP where there are differences in proton energy and, therefore, LET. There is also a lack of understanding of the DNA damage induced by PBT, particularly the complexity and relative levels of clustered/complex DNA damage (CDD) induced by protons at the distal edge of the Bragg peak. Consequently, the cellular DNA damage response (DDR) and repair pathways that are required for resolving CDD generated by PBT are not fully understood. Related to this, individual human cancers will furthermore display inherent differences in radiosensitivity to PBT, of which proteins involved in the DDR play such an important role. These uncertainties limit our ability to use PBT to its full advantage, by exploiting tumor killing while reducing the exposure of healthy tissue [3].

In this review, we provide the latest knowledge of the radiobiology of PBT, particularly in the context of DNA damage and the repair pathways that are important for the cellular DDR, and discuss the areas where ongoing research is necessary, which will have a major impact on the effective clinical use of PBT for cancer treatment.

## 2. Relative Biological Effectiveness (RBE) and Linear Energy Transfer (LET)

RBE is used to correlate PBT to photon radiotherapy, as is it the ratio of the reference radiation (photon) dose to the dose of protons required to cause the same biological effect. In clinical practice, a constant RBE value of 1.1 is utilized throughout the Bragg curve, despite the ongoing debate about whether this is the optimal solution or not [3,4,5]. RBE depends on both physical factors such as the proton beam energy, the dose fractionation and dose rate, and biological factors including the type of the tissue, cell-cycle stage, the oxygenation level, and the position of irradiation along the SOBP [5,6,7]. Experimental evidence largely derived from in vitro clonogenic survival assays using PBT facilities ranging from 65–250 MeV have demonstrated that the RBE value is variable and increases with decreasing dose [3,5,8,9]. In spite of the large fluctuation derived from in vitro data and the biological uncertainty, a constant RBE of 1.1 is used clinically to minimize the potential for risks [3,7,10,11]. One of the parameters mainly determining RBE values is the LET, which is the energy loss and deposition along the path of the proton beam and is a measure of ionization density [3,5]. Therefore, the higher the LET is, the denser the ionization events are, resulting in more extensive damage induction. High-energy PBT is considered low-LET irradiation; however, as the proton beam energy decreases throughout the SOBP, the LET increases particularly at the distal edge. Consequently, RBE values have been reported to rise from ~1.1 in the entrance, to ~1.2 in the center, ~1.4 at the distal edge, and ~1.7 in the distal fall-off the SOBP [11,12]. However, RBE values at the distal fall-off were shown to rise to over 3, which is supported by two other studies using clonogenic survival assays indicating RBE values of up to 2.3 [13] and 3.5 [14]. Furthermore, a dose shift around the distal edge where the biological dose extends beyond the range of the SOBP can threaten proximal healthy tissue, potentially causing unexpected side effects [15]. Interestingly a recent in vivo study using rat cervical spinal cords irradiated at four different positions of an SOBP demonstrated that RBE values varied from 1.1 to 1.3, dependent on LET [16]. The uncertainties and challenges with RBE are not covered at length here, and we refer the reader to the literature cited above and more recent reviews [17,18].

## 3. Radiobiological Effects of Protons

### 3.1. DNA Damage and Repair

**Figure 2:**
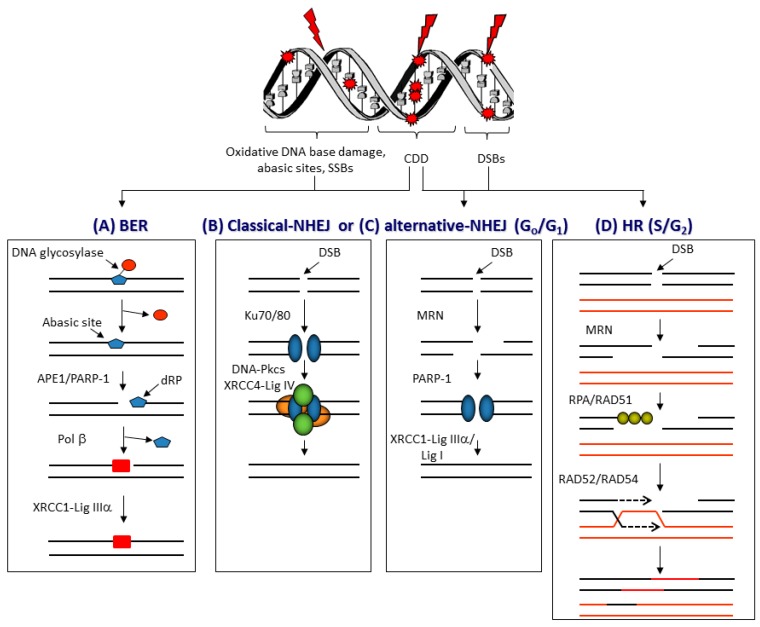
The response to ionising radiation (IR)-induced DNA damage. Proton beam therapy (PBT), similar to other radiotherapy techniques, targets DNA and can generate an abundance of DNA lesions, where oxidative DNA base damage, abasic sites, and single-strand breaks (SSBs) predominate, and which are repaired via (**A**) the base excision repair (BER) pathway. This involves recognition of the damaged base by a damage specific DNA glycosylase, incision of the abasic site by AP-endonuclease 1 (APE1) and SSB binding by poly(ADP-ribose) polymerase-1 (PARP-1), 5’-deoxyribosephosphate (dRP) removal and gap filling by DNA polymerase β (Pol β), and finally ligation by X-ray repair cross-complementing protein 1-DNA ligase IIIα (XRCC1–Lig IIIα) complex. Double-strand breaks (DSBs) are repaired by different pathways dependent on cell-cycle phase. In the G_0_/G_1_ phases, DSBs are repaired by either (**B**) classical non-homologous end-joining (NHEJ) involving Ku70/80 that binds to the DNA ends, followed by DNA-dependent protein kinase catalytic subunit (DNA-Pkcs) and XRCC4–Lig IV that promote DNA ligation, or via (**C**) alternative NHEJ which involves DSB end resection by the MRE11–RAD50–NBS1 (MRN) complex, PARP-1 binding to the DSB ends, and subsequent repair by Lig I or XRCC1–Lig IIIα. In the S/G_2_ phases of the cell cycle, DSB repair is achieved by (**D**) homologous recombination (HR) which uses a sister chromatid for repair. Therefore, following DNA end resection by the MRN complex, replication protein A (RPA) and RAD51 bind to the single-stranded DNA overhangs that promote strand invasion and subsequent DNA synthesis in the presence of RAD52/RAD54, as well as formation and resolving of Holliday junctions. The induction of complex DNA damage (CDD), consisting of several DNA lesions in close proximity, particularly by high-LET protons at the distal edge of the SOBP, likely require multiple pathways for repair.

The therapeutic effect of PBT, similar to conventional radiotherapy techniques, relies on significant DNA damage within tumorous cells leading to cell death. A variety of DNA lesions are induced along the radiation track (Figure 2), which include DNA base damage, sites of base loss (abasic sites), and DNA single-strand breaks (SSBs) that are most abundantly generated. On the other hand, the formation of DNA double-strand breaks (DSBs) and complex DNA damage (CDD) containing two or more DNA lesions in close proximity (within 1–2 helical turns of the DNA [19]) are less frequent, although these are considered the most lethal [20,21,22]. However, human cells have developed a sophisticated signaling network, the cellular DDR, which detects and repairs these DNA lesions [23]. DSBs are mainly resolved via two repair pathways, non-homologous end-joining (NHEJ) and homologous recombination (HR) (reviewed in [24,25]). Pathway choice is partly dependent on cell-cycle stage, with NHEJ mostly active in G_0_/G_1_, whereas HR is active in S/G_2_ phases [26]. NHEJ can be further divided into classical NHEJ, which involves the Ku70/80 heterodimer that binds to the DSB ends and recruits the DNA-dependent protein kinase catalytic subunit (DNA-Pkcs), and X-ray repair cross-complementing protein 4 (XRCC4)–DNA ligase IV that promotes the end-joining reaction (Figure 2B). Whereas alternative NHEJ involves DNA end resection by the MRE11–RAD50–NBS1 (MRN) complex, poly(ADP-ribose) polymerase-1 (PARP-1) that binds to the DNA ends, and X-ray repair cross-complementing protein 1-DNA ligase IIIα (XRCC1-Lig IIIα) or DNA ligase I (Lig I) that seals the DSB (Figure 2C). During HR, the DNA undergoes end resection by the MRN complex and the 3′-single stranded DNA is coated by replication protein A (RPA) and RAD51 that promotes invasion into the sister chromatid. DNA synthesis is followed by resolution of Holliday junctions before completing repair (Figure 2D). CDD, given that this contains localized damage over short distances within the DNA, can include a mixture of DNA base damage, abasic sites, SSBs, and DSBs [27]. This, therefore, represents a major barrier to the cellular DDR for efficient repair; however, considering the nature of the damage, it is assumed that these CDD sites will require the relevant proteins involved in base excision repair (BER), as well as DSB repair. BER is generally coordinated through the action of damage-specific DNA glycosylases that excise the damaged DNA bases, AP-endonuclease 1 (APE1) that incises the resulting abasic sites and generates an SSB for PARP-1 binding, DNA polymerase β (Pol β) that removes the 5′-deoxyribosephosphate moiety and inserts the correct undamaged nucleotide, and a complex of XRCC1–Lig IIIα that seals the SSB [28,29] (Figure 2A).

### 3.2. DNA Damage Induction and Repair Following PBT

Protons, as particles with mass and positive charge, interact with tissue completely differently from photons which have neither mass nor charge, although the specific physical aspects (e.g., beam intensity, LET, and secondary particle spectra) depend very much on the proton beam delivery system [30]. Consequently, DNA damage induction and the mechanisms of DNA repair employed are reportedly different between PBT and conventional radiotherapy [31]. Most of the focus of current studies is on examining the induction of DSBs, given that they are one of the major contributors, along with CDD, to cell lethality post-irradiation (Table 1). Firstly, a significantly higher level (~1.2–1.6-fold) of DSBs, particularly at 30 min post-irradiation, via analysis of phosphorylated histone variant H2AX (γH2AX) foci, was shown for a 200 MeV PBT source compared to 10 MV photons in two human tumor cell lines, ONS76 medulloblastoma cells and MOLT4 leukemia cells [32]. The disparities in foci number diminished after 6 h post-irradiation, although it was reported that the PBT-induced γH2AX foci in the ONS76 cells appeared to be ~1.2–1.5-fold larger in size, indicating a possible CDD phenotype. The fact that these foci were resolved with similar kinetics would, however, argue that these DSBs are possibly not complex in nature, given that CDD sites usually take a longer time to resolve. Similarly, the number of DSBs in SQ23B head and neck squamous cell carcinoma (HNSCC) cells measured by pulse-field gel electrophoresis was found to be ~1.2-fold higher for both 76 MeV and 201 MeV PBT sources than with photons induced by γ-irradiation [9]. Yet interestingly, DSB numbers were not significantly different between the two PBT energies or at different positions (entrance, mid, and distal) relative to the SOBP, and any potential differences in kinetics of DSB repair were not reported. Numbers of both DSBs and SSBs were also shown to be significantly higher (~1.2–1.6-fold increases in comet percentage tail DNA) in glioblastoma stem-like cells treated with protons in comparison to 320 kV X-rays, particularly at 20–48 h post-irradiation, which was associated with a higher level of apoptosis [33]. In contrast to the above studies, the numbers of γH2AX and 53BP1 foci (as DSB markers) induced in TrC1 prostate cancer cells and murine embryonic fibroblasts irradiated at the entrance dose of a 187 MeV PBT beam compared to 320 kV photons were observed to be the same 30 min post-irradiation [34]. The kinetics of DSB repair, specifically the resolving of γH2AX and 53BP1 foci, were also shown to be similar in response to the two irradiation conditions. This is supported by equal numbers of 53BP1 foci induced in AG01522 skin fibroblasts 30 min post-irradiation at the entrance dose, and their repair up to 24 h post-irradiation, of a 60 MeV proton beam compared to 225 kV X-rays [35]. Additionally, it was demonstrated that the initial level of induction of DSBs (γH2AX foci) was the same in wild-type, HR-deficient, and NHEJ-deficient Chinese hamster ovary cell lines following 1 Gy irradiation with low-LET 138 MeV PBT and 200 kV X-rays [36]. However PBT resulted in further reduced clonogenic survival in wild-type cell lines versus X-ray irradiation, suggesting that the quality of DNA damage (e.g., formation of CDD) is what differs between PBT and X-rays and their effectiveness in cell killing, although differences in levels of CDD was not proven directly.

Our recent study, using the neutral comet assay, demonstrated that the kinetics of repair of DSBs induced by the entrance dose of a proton beam (58 MeV) versus 100 kV X-rays in HeLa and HNSCC cells are not significantly different [37]. This would indicate that the nature and complexity of the DSBs following the two irradiation conditions are similar. Likewise, the kinetics of SSB/abasic site repair using the alkaline comet assay were comparatively the same. Furthermore, we observed that low-energy protons generated at the distal edge of a SOBP (11 MeV mean energy incident on the cells) had no impact on the repair of DSBs in both HeLa and HNSCC cells in comparison to 58 MeV protons and 100 kV X-rays, even though there was a significant difference in clonogenic survival between the proton irradiation conditions. However, we observed a significant delay in the repair of SSB/abasic sites only following low-energy proton irradiation. In fact, levels of SSBs were ~4–7-fold higher 2 h post-irradiation under these conditions, in comparison to cells irradiated with 58 MeV protons. This suggested that low-energy protons can generate CDD that is largely SSB-associated, which persists for several hours (>2 h) post-irradiation and contributes to decreased cell survival, although the specific nature and composition of the CDD under these conditions requires further research (see also Section 3.5).

### 3.3. Generation of Reactive Oxygen Species and Cell-Cycle Progression Following PBT

Related to DNA damage induction is the generation of reactive oxygen species (ROS). Interestingly, a more rapid and prominent increase in ROS following PBT was reported in neural precursor cells from rat hippocampus exposed to either 250 MeV protons near the Bragg peak, versus 250 kV X-rays [38]. Proton-induced ROS peaked 6 h post-irradiation and was ~1.5-fold above the control levels, while photon-induced ROS peaked 12 h post-irradiation and was ~1.3-fold above the control levels at a 5 Gy dose equivalent. However, less prominent increases and time-dependent differences in ROS levels were observed at a 1 Gy dose. Furthermore, it was shown that protons were more effective in killing cancer stem-like cells derived from non-small-cell lung cancer cell lines, and that compared to photons, protons induced higher levels (~1.1–1.7-fold) of ROS after treating these cells with equivalent doses of radiation [39]. ROS were also demonstrated to play an important role in inducing cytotoxicity of glioblastoma stem-like cells treated with protons, and ROS levels were ~1.8-fold higher following protons versus 320 kV X-rays at 20 h post-irradiation, which led to increased cellular apoptosis [33]. Levels of ROS were continually and dramatically higher (~6–7-fold) three days following protons in comparison to photons. 

Cell-cycle progression is another important factor related to proton-induced DNA damage, as DNA damage checkpoints will be activated to allow cells to undergo extensive DNA repair prior to DNA synthesis or replication. This is also important for understanding the repair pathway choice (see Section 3.4), given that DSBs can be repaired by either NHEJ or HR in different cell-cycle phases. One study conducted using human lung cancer cells suggested that, following 62 MeV protons, CRL5876 cells appeared to accumulate (~2-fold increase) in the G_1_ phase at 24 h post-irradiation, but that both CRL5876 and HTB177 cells accumulate (~1.5–2-fold increase) in G_2_/M at 48 h post-irradiation, versus unirradiated controls [40]. However, no comparisons against photon irradiation were performed. We also recently noted an accumulation (~1.5-fold increase) of HeLa cells in G_2_/M, particularly at 8–24 h post-irradiation with 58 MeV PBT, which was not LET-dependent as the same observation was seen with cells irradiated with low-energy protons generated at the distal edge of a SOBP (11 MeV mean energy incident on the cells) at higher LET [37,41]. This, however, suggests that CDD induced by high-LET protons, at least under the conditions analyzed, is not a major contributory factor to the observed cell-cycle checkpoint activation. An early study observed G_2_ arrest of glioblastoma cells at 24–72 h post-irradiation following 5.7 MeV protons at relatively high-LET, which was more pronounced (~1.5–2.5-fold) than irradiation of cells using 120 kV X-rays [42], suggesting potential proton-specific effects. In contrast, there was no dramatic difference in cell-cycle distribution of Chinese hamster ovary cells when comparing the response to low-LET 138 MeV PBT and 200 keV X-rays, where a degree of G_2_/M accumulation (~1.2–2-fold increase) of cells irradiated at 5 Gy dose equivalent, particularly at 6–12 h following both irradiation types, was observed [36]. Furthermore, it was shown that proton irradiation of glioblastoma stem-like cells actually led to a shortened G_2_/M arrest compared to 320 kV X-ray irradiation, as demonstrated by a ~2-fold accumulation of cells in this cell-cycle phase at six days post-irradiation following photon irradiation only [33]. However, the baseline levels of cells in G_2_/M in this study were noticeably different (~10 and 20 %) in the experiments comparing proton and photon irradiation. Given the variability in the observations, more studies to directly compare progression of cells through the cell cycle in response to protons versus photons in specific cell models, and the impact of LET need to be performed.

### 3.4. DSB Repair Pathway Choice Following PBT 

NHEJ is considered the primary mechanism for DSB repair, particularly in response to photon irradiation, but there are a few conflicting reports to date suggesting that the DNA repair pathway choice specifically following PBT may in fact be different (Table 2). Firstly, by studying DNA repair kinetics in wild-type, NHEJ-deficient (XRCC4 and DNA-Pkcs) and HR-deficient (XRCC2 and XRCC3) Chinese hamster cell lines exposed to photon (γ-irradiation) versus low-LET 200 MeV protons, the same biological effect was observed in each cell line comparing the two radiation types [43]. Therefore, a delayed decrease in γH2AX foci at 3–12 h post-irradiation, as well as significantly reduced clonogenic survival, was observed in DNA-Pkcs-deficient cells in comparison to wild-type cells. However, HR-deficient cells also displayed increased sensitivity to protons and photons, and significantly higher chromosomal aberrations (~2–4-fold increases) were found in both NHEJ- and HR-deficient cells compared to the wild-type cells following both radiation types. From this study, it was suggested that NHEJ is the major pathway, and DNA-Pkcs is the main protein involved in resolving DSBs induced not only by photons but also by low-LET protons. This is supported by another study demonstrating that there were no significant differences in γH2AX foci formation and their repair in wild-type and DNA-Pkcs-deficient Chinese hamster ovary cell lines in response to γ-irradiation versus low-LET protons [44]. Persistent γH2AX foci was observed in the DNA-Pkcs-deficient cells 6 h post-irradiation with higher doses (2-3 Gy) of photons or protons, correlating with increased radiosensitivity versus wild type cells. In contrast using a similar experimental set-up of Chinese hamster ovary cell lines deficient in HR (XRCC3) or treated with small interfering RNA (siRNA) targeting RAD51 and comparing low-LET 138 MeV proton and 200 kV photons, it was suggested that PBT induced more lethal chromosomal aberrations [36]. Moreover, this study reported that PBT was more effective in killing HR-deficient cell lines than NHEJ-deficient cells and, therefore, there was an enhanced dependence on HR for repair of proton-induced DSBs. The same conclusion was found following an examination of low-LET PBT (138 MeV) versus 200 kV photon irradiation in human tumor cells that were treated with siRNA or inhibitors targeting key proteins involved in HR and NHEJ [45]. It was found that DNA-Pkcs inhibition significantly radiosensitized A549 lung cancer and glioblastoma cells to photon-irradiated cells, but that this was to a lesser degree following low-LET PBT. Photon-irradiated cells in the presence of the inhibitor also showed delayed resolving of γH2AX foci at 6–24 h post-irradiation which were ~1.5–3-fold higher than the corresponding cells following proton irradiation. In addition, it was found that HR-deficient cell lines (RAD51 siRNA) were more sensitive to proton irradiation and similarly had difficulty resolving γH2AX foci, again suggesting a dependence of the cells to utilize HR for repairing proton-induced lesions. Evidence examining the response of HeLa cells to 21 MeV protons by immunostaining and high-resolution microscopy demonstrated an association of RAD51 with almost every 53BP1 foci 1 h post-irradiation, also indicating that the proportion of cells undergoing HR following PBT may be higher [46]. Interestingly, when examining the comparative RBE of 17 non-small-cell lung cancer cell lines in response to 235 MeV protons and 250 kV X-rays, only five of these displayed increased sensitivity to protons and two had confirmed defects in *BRCA1* indicative of a deficiency in HR [47]. The unexpected differences in RBE between protons and photons was again predicted to be due to differences in the formation of CDD. Given these opposing findings, more definitive evidence of the DNA repair pathway choice following proton irradiation is necessary.

### 3.5. CDD Formation Following PBT

Given the increase in LET toward the distal edge of the SOBP, this is considered to be particularly effective in increasing the amount of CDD, which is similar in nature to that observed following heavy-ion irradiation [31]. CDD is considered equally as effective as DSBs in cell killing due to the difficult nature of its repair leading to its persistence in cells and tissues [27]; therefore, it should be considered as a crucial factor in the cellular response to PBT. However, to date, most of the evidence relating to CDD formation specifically following proton irradiation is indirect. Indeed, through Monte Carlo simulations, and by examining DNA damage clustering with increasing PBT energies (500 keV–50 MeV) and, thus, decreasing LET, the amount and size of both complex SSBs and complex DSBs were found to decrease [48]. Similarly, the relative frequencies of complex SSBs and DSBs were also shown to increase proportionally with increasing LET, which is dependent on proton energy [49,50]. A biophysical model of radiation-induced cell death and chromosomal aberrations based on the critical role of CDD, and compared to experimental data in AG01522 and V79 cells following irradiation with 62 MeV protons predicted that these end-points increased along the SOBP and were highest at the distal fall-off due to low-energy protons [6]. Cell death at a 2 Gy dose was calculated to increase ~1.5-fold and chromosome aberrations (dicentrics per cell) increased ~4-fold at the distal fall-off compared to the entrance dose. Additionally, more recently, Monte Carlo simulations were utilized to examine unrepaired DSBs 24 h after proton irradiation, which were observed to increase ~1.5-fold (2 Gy) and 1.7-fold (5 Gy) toward the distal fall-off of the SOBP at higher LET, predictably through increased DSB complexity [51].

In relation to experimental evidence, apart from observations of changes in RBE via clonogenic survival assays, which are suggestive of CDD formation, direct evidence is lacking, as CDD is notoriously difficult to measure and specifically define in terms of the nature of DNA damage complexity in vivo [21,52]. However in SQ23B HNSCC cells CDD, specifically complex DSBs measured by utilizing the *Escherichia coli* enzymes Fpg and Nth for excision of residual oxidative DNA base damage prior to pulse-field gel electrophoresis, was found to be ~1.2-fold higher for PBT at 76 MeV, but not 201 MeV, in comparison to γ-irradiation [9]. Interestingly, CDD formation did not depend on the position of irradiation in the SOBP, which conflicts with other reported data. In particular, it was demonstrated in AG01522 skin fibroblasts that persistent 53BP1 foci, as a marker of DSBs, was evident when cells were irradiated at the distal end of the SOBP of a 60 MeV proton beam in comparison to cells irradiated at the entrance dose or at the Bragg peak itself [35]. These persistent foci were evident at 24 h post-irradiation with Bragg peak protons and were elevated ~2-fold in comparison to the entrance dose and to 225 kV X-ray irradiation. This is supported by observations of a delay in resolving γH2AX and 53BP1 foci in TrC1 prostate cancer cells and murine embryonic fibroblasts irradiated at the Bragg peak (31 MeV) compared to those irradiated at the entrance dose (187 MeV) [34]. Whilst the initial numbers of γH2AX and 53BP1 foci under the comparative conditions were observed to be the same, there were significantly (~1.1–1.3-fold) higher levels of foci particularly at 1–4 h post-irradiation in cells irradiated at the Bragg peak, and these foci were also shown to be on average ~1.3-fold larger in size at 0.5 h and 6 h post-irradiation. However, all foci, indicative of DSB levels, were shown to be resolved by 24 h irrespective of the irradiation set-up. These two studies are suggestive of the formation of complex DSBs, particularly at higher LET, which have a longer lifetime to resolve, although direct evidence for this was not presented. More recently, we described utilization of different versions of the comet assay to directly demonstrate that CDD is generated in HeLa and HNSCC cells by low-energy protons (11 MeV mean energy incident on the cells; relatively high-LET) at the distal edge of an SOBP, in comparison to the cells irradiated at the entrance of a proton beam (58 MeV mean energy; low LET) that do not [37]. In particular, using an alkaline version of the comet assay, we showed that low-energy protons caused a reduced rate of repair of cellular SSBs and alkali-labile sites, suggesting that CDD was largely SSB/abasic site in nature. Under these conditions, we observed that SSB levels in cells were ~4–7-fold higher 2 h post-irradiation in comparison to cells irradiated with 58 MeV protons. Interestingly, there was no defect in the repair of DSBs visualized using the neutral comet assay. Furthermore, an enzyme-modified neutral comet assay employing recombinant DNA repair enzymes to excise any residual oxidative DNA base damage and abasic sites in association with DSBs confirmed direct evidence that CDD is formed by low-energy protons generated at the distal end of the SOBP. We demonstrated that CDD formation in HeLa cells was increased by ~1.3-fold immediately post-irradiation with low-energy protons versus 58 MeV protons, and that this damage persisted for at least 4 h post-irradiation. These findings altogether highlight the ability of PBT to induce potentially more lethal CDD at and around the Bragg peak where the highest LET occurs.

### 3.6. Cellular Response to CDD Generated by PBT

**Figure 3:**
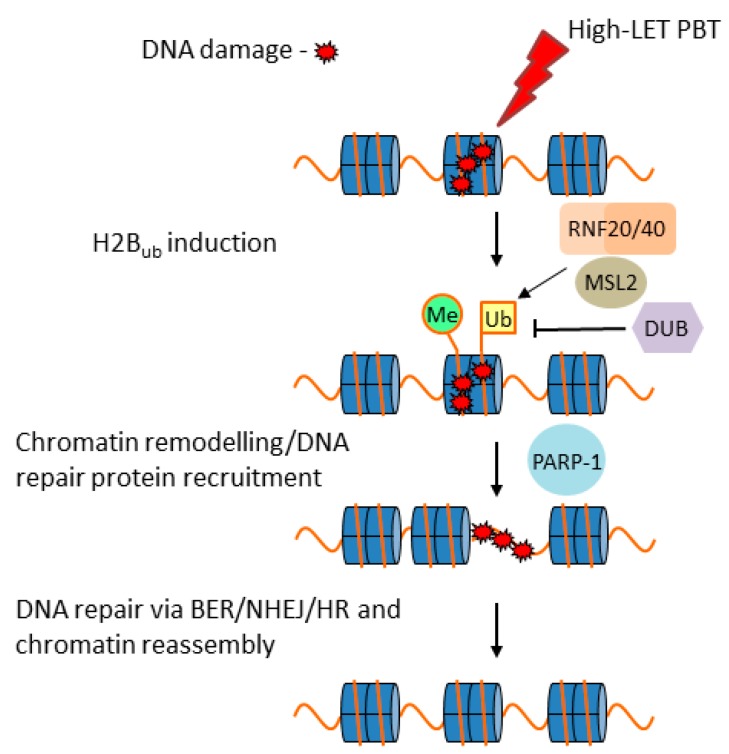
Proposed model for the cellular response to complex DNA damage (CDD) induced by proton beam therapy (PBT) in chromatin. On induction of CDD, this triggers monoubiquitylation of histone H2B on lysine 120 (Ub) by the E3 ubiquitin ligases ring finger 20/40 complex (RNF20/40) and male-specific lethal 2 homolog (MSL2). This stimulates recruitment of the necessary DNA repair proteins and/or chromatin remodeling factors that promote CDD accessibility. Poly(ADP-ribose) polymerase-1 (PARP-1) in particular is essential for efficient CDD repair. Our evidence also suggests the involvement of histone trimethylation (Me) and predictably a deubiquitylation enzyme (DUB) that is able to regulate access to CDD. Repair then proceeds through the respective DNA repair pathway dependent on the nature of the damage, although we suggest a particular dependence on the base excision repair (BER) pathway in the cellular response to high-LET protons, prior to subsequent chromatin assembly.

CDD sites are considered lethal, although this very much depends on the degree of complexity and the nature of the damage. Indeed, given that, broadly speaking, these are likely to consist of either complex SSBs or complex DSBs, the cellular response to these may require multiple DNA repair pathways and proteins, including, as indicated above (Figure 2), a combination of BER and NHEJ/HR [22,27]. However, despite an appreciation that CDD is a critical factor in the radiobiology of PBT, the cellular response to CDD induced by PBT, particularly with increasing LET along the SOBP, is surprisingly understudied. Predictably, there should be a signaling (DDR) mechanism within cells, similar to γH2AX for DSBs, which is responsible for promoting the repair of CDD sites. We recently reported for the first time that monoubiquitylation of lysine 120 on histone H2B is promoted in HeLa and HNSCC cells in response to CDD induced by low-energy (11 MeV mean energy incident on the cells) protons at the distal edge of an SOBP, catalyzed by the E3 ubiquitin ligases ring finger 20/40 complex (RNF20/40) and male-specific lethal 2 homolog (MSL2) [37]. In fact, levels of histone H2B ubiquitylation increased by ~1.3–1.6-fold in HeLa cells and ~1.6–2.2-fold in HNSCC cells at 3–6 h post-irradiation. We demonstrated that this mechanism is important for the efficient repair of CDD sites, as revealed by delayed repair and significant persistence of CDD induced by low-energy protons in RNF20/40 and MSL2 siRNA-depleted cells using the enzyme-modified neutral comet assay, where CDD levels were ~2.3-fold higher compared to the non-targeting control siRNA treated cells at 4 h post-irradiation. Furthermore, RNF20/40 and MSL2 were shown to be required for promoting cell survival under these conditions, as revealed by clonogenic assays. We, therefore, believe that this is a mechanism for signaling recruitment of DNA repair proteins and/or for chromatin remodeling necessary for CDD repair (Figure 3). We also described possible evidence that other chromatin changes, particularly through histone trimethylation, are evident following irradiation of cells with low-energy protons; however, whether this is directly related to CDD repair is currently unknown. As a development of these findings, we also recently performed siRNA screening of deubiquitylation enzymes (DUBs) to further identify the specific enzymes controlling protein ubiquitylation that are involved in modulating cell survival in response to CDD induced by low energy (11 MeV; relatively high LET) protons at the distal edge of an SOBP, versus more simple DNA damage generated by both low-LET (58 MeV) protons and 100 kV X-ray irradiation [41]. This study revealed that ubiquitin-specific protease 6 (USP6) is required to promote survival in HeLa and HNSCC cells specifically in response to low-energy protons, and that this effect is mediated through stabilization of the SSB repair protein PARP-1 required for efficient CDD repair. In fact, levels of CDD were ~1.8-fold higher in USP6 siRNA-depleted cells compared to the non-targeting control siRNA treated cells at 4 h post-irradiation. This evidence was strengthened and mimicked using the PARP inhibitor olaparib, or through depletion of PARP-1 using siRNA, which was demonstrated to increase the radiosensitivity of cells to low-energy protons as a consequence of a significant deficiency in CDD repair. This correlates with our previous evidence suggesting that CDD generated under these conditions is largely SSB in nature [37], and that PARP-1 plays a critical role in its repair. However, our study revealed significant synergy between PARP inhibition and CDD induced by low-energy protons in enhancing cancer cell killing. Predictably, there is also dependence on other proteins in the BER pathway (such as APE1, Pol β, and XRCC1–Lig IIIα; Figure 2A) required to promote CDD repair.

Previous studies of CDD have largely focused on high-LET heavy-ion irradiations. Here, these have demonstrated that in irradiated cells, CDD increases with increasing LET, but that these are predominantly unrepairable CDD that generate either chromosome aberrations through the lack of cell-cycle checkpoint activation or drive cells into senescence [53,54]. Therefore, it is important not to draw direct parallels between the unrepairable, highly complex CDD generated by heavy ions, and CDD sites generated by PBT which are likely to be less complex in nature and indeed repairable. Furthermore, it is thought that CDD may also be prone to generating increases in mutation frequency due to abortive or slow repair of CDD sites [55]. Nevertheless, due to technical limitations and lack of experimental studies in this area, we do not have a full appreciation of the cellular response to CDD specifically generated by PBT at different energies along the radiation track, and whether the nature of the damage, particularly toward the distal edge, is of sufficient complexity to drive mutagenesis and/or chromosomal aberrations. Therefore, more extensive research in this area is necessary.

## 4. Conclusions and Outlook

The utilization of PBT for cancer treatment is increasing worldwide and is appreciated to be advantageous over conventional radiotherapy as the maximum energy deposition occurs in a well-defined region (the Bragg peak) that can be specifically targeted to the tumor, which minimizes unnecessary irradiation of the surrounding normal tissues and OAR. However, there are still uncertainties with the radiobiology of PBT along the track of the proton beam and particularly the generation of high-LET protons at the distal edge that can have a greater impact on the molecular and cellular effects. Therefore, there is an urgent need to further understand the biological effects of PBT, and particularly to understand the impact on DNA and how this varies with LET. Indeed, whilst it is widely accepted that CDD is induced at the distal edge of the SOBP, there is little information on the nature of the damage (e.g., DSB- or SSB-associated) related to proton energy/LET, and how cells are able to process this through cellular DDR pathways. This is challenging given that CDD is difficult to measure in vivo; thus, new strategies need to be devised to tackle this problem. There is also conflicting evidence that simple DSBs induced by PBT are largely repaired by HR, in contrast to NHEJ which is employed in response to photon irradiation, and whether this is cell-type-dependent. Furthermore, there are potential differences in the levels of ROS and impact of cell-cycle progression between protons and photons, although again more experimental data are required to substantiate these findings. These essential studies have to be carefully designed, particularly as cancer cell lines frequently have defects in DNA repair and in the cellular DDR; furthermore, irradiation of cells in specific cell-cycle phases must be taken into account given the dependence of cells to largely utilize HR in S/G_2_ phases.

Another consideration is that additional experimental models and techniques need to be utilized in PBT research, rather than the conventional in vitro experiments using cultured monolayer cell lines mostly used to assess clonogenic survival post-irradiation. Increasingly, three-dimensional (3D) models are being employed in translational research, which more accurately reflect the structure and environment of the original tumor. Therefore, either 3D spheroid models of cancer cell lines, or multicellular spheroids encapsulating the tumor cells within the correct cellular microenvironment should be used to examine spheroid growth in response to PBT. These models will also allow a further examination of PBT radiobiology in terms of the types of DNA damage (e.g., DSBs and CDD) induced throughout the SOBP, the DNA repair pathways essential for their repair, and the impact of combinations of targeted drugs or inhibitors (e.g., those targeting the DDR [56]) with PBT in effective suppression of 3D spheroid growth. The next level would be to employ patient-derived organoids for examining how these respond to PBT in vitro, and possibly in the future to use these as predictive models for determining tumor response and ultimately patient outcome to PBT. Finally, more in vivo experiments employing xenograft models to assess growth of specific tumors following PBT, such as those conducted using HNSCC [57], should be conducted. These additional models and experiments bring their challenges, such as the availability and use of clinical facilities for performing animal irradiations, and technical challenges including the precise positioning and delivery of PBT to animals. In addition, a large proportion of PBT facilities worldwide are not usually equipped with on-site laboratories to effectively perform biological experiments in vitro and in vivo.

There is also an added level of complexity in terms of considering biological factors that may have a significant impact on the cellular DDR to PBT, particularly on overall efficacy of the treatment. For example, tumor hypoxia is well known to represent a barrier to the effectiveness of photon radiotherapy, although there is evidence that particle therapy with higher LET, particularly carbon ions, has a lower oxygen enhancement ratio and can, therefore, overcome radioresistance of the tumors. However, whether PBT, particularly at the Bragg peak and the associated distal edge with higher LET, is able to have the same impact on experimental models is unclear. Also, the tumor microenvironment is of particular importance given the recent success of immune checkpoint inhibitors (e.g., targeting PD-1/PD-L1) and their effective combination with radiotherapy for cancer treatment. However, again, there is little evidence available to understand the added benefit of immunotherapy strategies in combination with PBT. Nevertheless, there should be a drive from the clinical and scientific community to collaborate and engage in driving this preclinical and translational research which will ultimately be utilized for the optimization and personalization of PBT for patient benefit.

In summary, future PBT research should focus on the following:

- Further understanding of the biological effect of PBT at different energy/LET on the cellular DDR;
- Employing additional in vitro models (e.g., 3D spheroids/organoids) in radiobiology experiments;
- Increased utilization of in vivo experiments employing specific tumor models;
- Consideration of other biological factors (e.g., hypoxia, tumor microenvironment).

## Figures and Tables

**Table 1:** Comparisons of double-strand breaks (DSBs) induced by proton beam therapy (PBT) versus photon irradiation.

Cell Line	Method(s)	Proton Energy	Photon Energy	Observation (Proton vs. Photon)	Ref
ONS76 medulloblastoma; MOLT4 leukemia cells	γH2AX foci by immunofluorescence	200 MeV	10 MV X-rays	~1.2–1.6-fold increase in DSB foci and ~1.2–1.5-fold larger in size 30–180 min post-irradiation	[32]
HeLa; SQ20B HNSCC cells	Pulse-field gel electrophoresis	76 MeV, 201 MeV	622 keV ^137^Cs γ-rays	~1.2-fold increase in DSBs. No differences between PBT energies, nor along the SOBP	[9]
IN528 and T4213 glioblastoma stem-like cells	Alkaline and neutral comet assay	N.S.	320 kV X-rays	~1.2–1.6-fold higher numbers of DSBs at 20–48 h post-irradiation	[33]
TrC1 prostate cancer cells; murine embryonic fibroblasts	Histone γH2AX and 53BP1 foci by immunofluorescence	187 MeV entrance dose	320 kV X-rays	Similar numbers of DSBs at 0.5–24 h post-irradiation	[34]
AG01522 skin fibroblasts	53BP1 foci by immunofluorescence	60 MeV entrance dose	225 kV X-rays	Similar numbers of DSBs at 0.5–24 h post-irradiation	[35]
Wild-type, HR-, and NHEJ-deficient Chinese hamster ovary cell lines	Histone γH2AX foci by immunofluorescence	138 MeV	200 kV X-rays	Similar initial induction of DSBs	[36]
HeLa; UMSCC74A and UMSCC6 HNSCC cells	Neutral comet assay	58 MeV entrance dose; 11 MeV distal edge	100 kV X-rays	No difference in DSB repair kinetics	[37]

N.S. refers to not specified. HR—homologous recombination; NHEJ—non-homologous end-joining; SOBP—spread-out Bragg peak.

**Table 2:** DNA double strand break (DSB) repair pathway choice following proton beam therapy (PBT) versus photon irradiation.

Cell line	Irradiations	Outcome	Ref
Wild-type, HR- and NHEJ-deficient Chinese hamster ovary cell lines	200 MeV protons and ^137^Cs γ-rays	NHEJ is the major pathway for both photons and low-LET protons	[43]
Wild-type and NHEJ-deficient Chinese hamster ovary cell lines	14.4 MeV plateau protons and 667 keV ^137^Cs γ-rays	NHEJ is the major pathway for both photons and low-LET protons	[44]
Wild-type, HR-, and NHEJ-deficient Chinese hamster ovary cell lines	138 MeV protons and 200-kV X-rays	Dependence on HR following protons	[36]
A549 lung cancer; glioblastoma cells	138 MeV protons and 200 kV X-rays	Dependence on HR following protons	[45]
HeLa	21 MeV protons	Higher proportion of cells undergoing HR following protons	[46]
Non-small-cell lung cancer cells	235 MeV protons and 250 kV X-rays	HR only partly required following protons	[47]

LET—linear energy transfer; HR—homologous recombination; NHEJ—non-homologous end-joining.
